# Biosafety, biosecurity and antimicrobial resistance containment in Kenya: A One Health scoping review of national and subnational systems

**DOI:** 10.1371/journal.pgph.0006477

**Published:** 2026-08-26

**Authors:** Lazarus Okayo Odeny, Leonard Kingwara, Kennedy Yatich, Benson Ulo, Maureen Kimenye, Jane Owenga

**Affiliations:** 1 Amref Health Africa in Kenya, Nairobi, Kenya; 2 Kenya National Public Health Institute, Nairobi, Kenya; 3 Jaramogi Oginga Odinga University of Science and Technology, Bondo, Kenya; Universitas Muhammadiyah Aceh, INDONESIA

## Abstract

Antimicrobial resistance (AMR) is a growing global health threat requiring robust biosafety, biosecurity, and surveillance systems across human, animal, and environmental sectors. However, evidence on how these systems function at sub-national and facility levels remains limited, particularly in decentralized settings such as Kenya. This scoping review was conducted in line with PRISMA-ScR reporting standards to map evidence on AMR containment systems in Kenya. Peer-reviewed and grey literature covering governance, surveillance, laboratory capacity, workforce, biosafety and biosecurity, digital systems, and One Health integration between January 2022 - February 2026 were systematically identified and thematically synthesized, with methodological quality assessed using the Mixed Methods Appraisal Tool. A total of 31 studies (23 peer-reviewed and 8 grey literature) were included. Human health systems were the most frequently represented domain (n = 27), followed by data integration and digital platforms (n = 16) and animal health (n = 14). Governance and policy-related components were reported in fewer studies (n = 10), while environmental health was the least represented (n = 8). Despite strong national policies and digital platforms, implementation was uneven across counties and facility levels, with key constraints including workforce shortages, infrastructure gaps, fragmented financing, and limited use of surveillance data. These findings highlight persistent gaps in sub-national implementation and One Health integration that limit the effectiveness of AMR containment systems in Kenya.

## Introduction

The effectiveness of modern antimicrobial therapy is increasingly compromised by the global rise of antimicrobial resistance (AMR), which contributes substantially to preventable morbidity, mortality, and health system strain [1]. The World Health Organization (WHO) has identified AMR as one of the major global public health threats requiring coordinated multisectoral action through strengthened surveillance, infection prevention, antimicrobial stewardship, and One Health approaches [1]. Global surveillance reports continue to demonstrate increasing resistance across priority bacterial pathogens and substantial gaps in AMR surveillance capacity, particularly in low- and middle-income countries (LMICs) [2]. Recent global estimates indicate that bacterial AMR was associated with approximately 4.95 million deaths in 2019, including 1.27 million deaths directly attributable to AMR, with the highest burden occurring in LMICs, particularly in sub-Saharan Africa [3]. Addressing AMR demands coordinated action across human, animal, and environmental health systems, reflecting the interconnected drivers of resistance at the human–animal–environment interface. Consequently, global strategies increasingly emphasize a One Health approach that integrates biosafety, biosecurity, surveillance, and antimicrobial stewardship across sectors [4].

In response, many countries have developed National Action Plans for AMR (NAP-AMRs), aligned with global frameworks such as the World Health Organization (WHO) Global Action Plan on AMR [1], the International Health Regulations, and the Joint External Evaluation process. These frameworks provide strategic guidance for governance, surveillance, infection prevention, and responsible antimicrobial use. However, evidence increasingly shows that the effectiveness of AMR policies depends not only on their adoption but also on their operationalization at sub-national and facility levels, where health services are delivered and surveillance data are generated. This challenge is particularly pronounced in decentralized health systems, where variability in resources, workforce capacity, and infrastructure can substantially influence system performance.

Within this global and regional context, Kenya has made notable progress in establishing national frameworks for AMR containment and multisectoral coordination. Efforts were formalized through the National Policy on Prevention and Containment of AMR and operationalized via the first National Action Plan (2017–2022) [5]. These were complemented by the national AMR Surveillance Strategy, which established laboratory-based surveillance of priority pathogens and alignment with the WHO Global Antimicrobial Resistance and Use Surveillance System (GLASS).

AMR surveillance is coordinated through the National Public Health Laboratories and overseen by the National Antimicrobial Stewardship Interagency Committee [5–7]. Initial implementation through sentinel sites revealed important gaps in laboratory information systems, data quality, representativeness, and integration. Subsequent investments led to the establishment of a centralized AMR data repository and the development of a One Health AMR Surveillance System to support national-level data integration across human and animal health sectors. Parallel initiatives have strengthened surveillance in the animal health sector, although routine environmental AMR surveillance remains limited.

Despite strong national policy and surveillance foundations, implementation challenges persist, particularly at sub-national and facility levels [6]. Under Kenya’s devolved governance structure, counties are responsible for service delivery, laboratory operations, and workforce management, resulting in substantial heterogeneity in AMR system readiness across regions and facility tiers. Variability in laboratory infrastructure, biosafety and biosecurity practices, workforce capacity, surveillance coverage, and data quality may undermine the reliability and public health utility of AMR surveillance outputs [2,6].

Similar patterns have been reported across sub-Saharan Africa, where AMR surveillance is often concentrated in higher-level urban facilities, limiting representativeness and early detection of emerging resistance [8,9]. While investments in digital systems and genomic capacity have strengthened national-level capabilities, the translation of surveillance data into routine infection prevention, antimicrobial stewardship, and policy action at county and facility levels remains inconsistent. The revised National Action Plan on AMR (2023–2027) explicitly highlights these challenges and calls for strengthened county-level governance, laboratory networks, workforce capacity, infection prevention and control (IPC), environmental surveillance, and sustainable financing within a One Health framework [7,10].

Although a growing body of research exists on AMR containment in Kenya—including laboratory capacity assessments, surveillance evaluations, policy analyses, and digital health interventions—this evidence remains fragmented across sectors and disciplines [2,9,11]. There remains limited synthesis of how biosafety, biosecurity, surveillance, and stewardship systems interact across national, sub-national, and facility levels, and how these influence implementation within a One Health framework [8,10].

This scoping review therefore sought to systematically map and synthesize evidence on biosafety, biosecurity, and AMR containment systems in Kenya. Specifically, the review addressed the following research question: How are AMR containment systems—including biosafety, biosecurity, and surveillance—assessed and implemented across national, sub-national, and facility levels in Kenya, and what are the key gaps, enabling factors, and evidence needs within a One Health framework?

By consolidating evidence across sectors and system levels, this review provides a comprehensive understanding of the operational performance of AMR containment systems in Kenya. The findings are intended to inform policy implementation, strengthen system integration, and identify priority areas for future research. Given the similarities in health system challenges across many LMICs, the insights generated may also be relevant to other decentralized settings seeking to strengthen integrated AMR surveillance and containment systems.

## Methods

### Study design

This study employed a scoping review design to systematically map and synthesize evidence on biosafety, biosecurity, and AMR containment systems in Kenya and comparable settings. The review followed the methodological framework proposed by Arksey and O’Malley and further refined by Levac et al. [12,13]. Reporting was conducted in accordance with the Preferred Reporting Items for Systematic Reviews and Meta-Analyses extension for Scoping Reviews (PRISMA-ScR) [14].

### Ethics statement

Ethical approval for this study was obtained from the Amref Health Africa Ethics and Scientific Review Committee (ESRC; Approval No. P2009/2025) and the National Commission for Science, Technology and Innovation (NACOSTI; License No. NACOSTI/P/26/4185647).

Although this study involved secondary analysis of publicly available data and published literature, ethical clearance was sought to ensure compliance with national research regulations and institutional requirements. The study adhered to principles of research integrity, including accurate representation of findings, proper citation of sources, and transparency in reporting.

### Eligibility criteria

Studies were included if they assessed biosafety, biosecurity, or AMR containment systems at national, sub-national, or facility levels within human, animal, or environmental health sectors. Eligible studies examined system components including governance and policy frameworks, laboratory capacity, workforce readiness, surveillance systems, antimicrobial stewardship, IPC, or implementation of AMR-related policies.

Quantitative, qualitative, and mixed-methods studies, as well as implementation-focused assessments, were included. Studies conducted in Kenya were prioritized, while regional and global studies were included where they provided contextually relevant evidence for Kenya or similar LMIC settings.

Studies were excluded if they were editorials, commentaries, opinion pieces, narrative reviews without systematic methods, or lacked primary or systematically analysed secondary data.

### Information sources and search strategy

A comprehensive search strategy was developed to identify peer-reviewed and grey literature. Databases searched included PubMed, Google Scholar, OpenAlex, and Semantic Scholar. Additional records were identified through citation chaining and reference list screening.

Grey literature was sourced from institutional websites, including the World Health Organization (WHO), Food and Agriculture Organization (FAO), World Organisation for Animal Health (WOAH), United Nations Environment Programme (UNEP), Africa CDC, and relevant Kenyan government institutions.

Search terms included combinations of keywords and controlled vocabulary related to AMR, biosafety, biosecurity, laboratory capacity, surveillance systems, antimicrobial stewardship, IPC, and One Health. Boolean operators (“AND,” “OR”) were used to refine searches. An example search string was:

(“antimicrobial resistance” OR AMR) AND (biosafety OR biosecurity OR surveillance OR antimicrobial stewardship OR “One Health”) AND Kenya.

Search strings were adapted to the indexing requirements and search functionalities of each database. Searches were limited to English-language publications published between January 2022 and February 2026.

### Study selection

Study selection followed a multi-stage screening process in accordance with PRISMA-ScR standards. Following database searches and identification of grey literature sources, all retrieved records were imported into a screening database and duplicates removed. Titles and abstracts were independently screened by two reviewers against predefined eligibility criteria. Potentially relevant records underwent full-text review to determine final inclusion.

**Fig 1:**
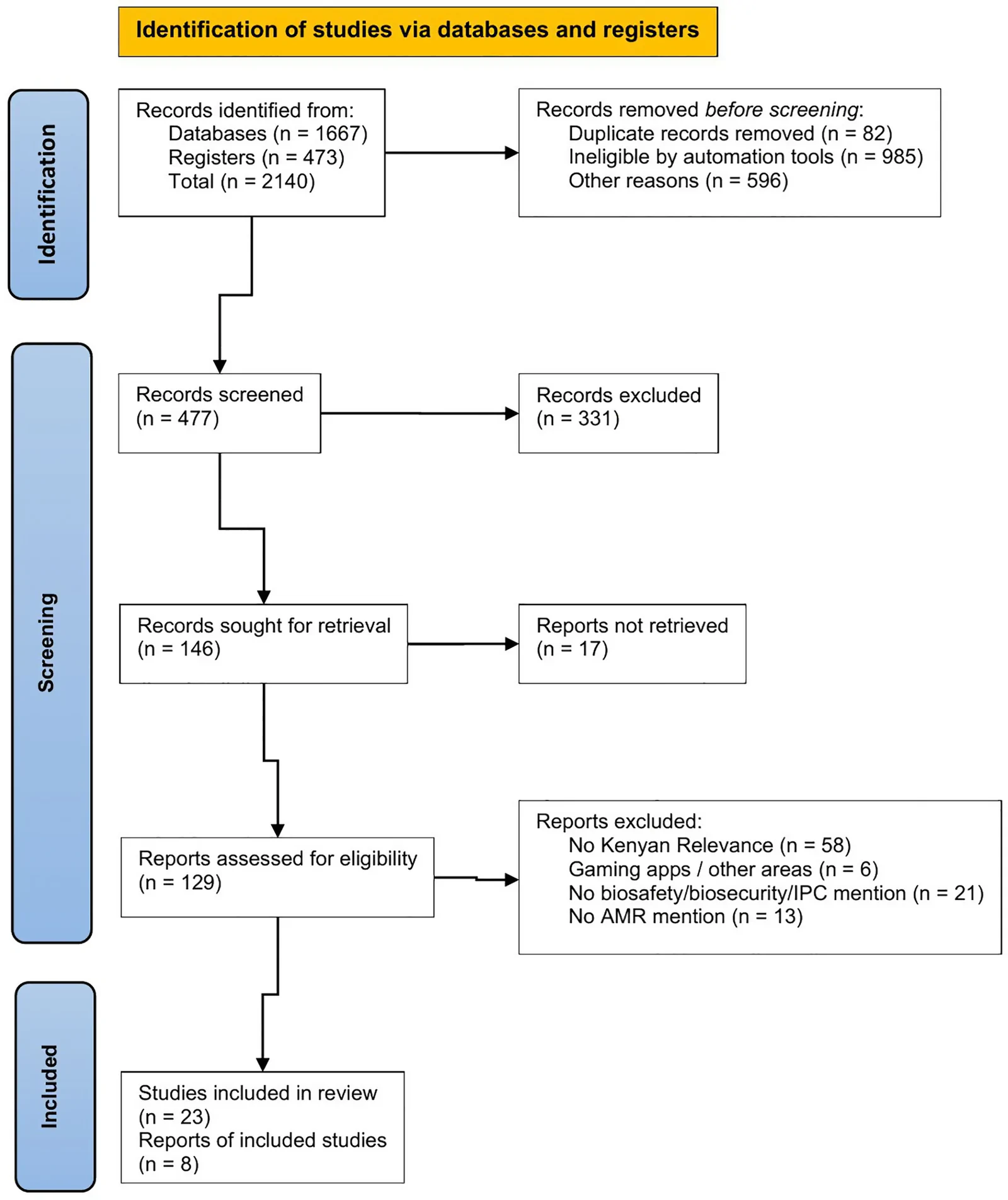
PRISMA-ScR flow diagram of study selection.

Disagreements arising during screening or full-text assessment were resolved through discussion and consensus between reviewers. Reasons for exclusion at the full-text stage were documented. The study selection process was summarized in the PRISMA-ScR flow diagram (Fig 1).

### Data extraction

A standardized data extraction tool was developed and pilot-tested on a subset of included studies and refined before full data extraction to ensure consistency. Two reviewers independently extracted data on study characteristics (author, year, setting), study design, sectoral focus, system components assessed, frameworks used, and key findings. Additional data were collected on One Health integration, implementation barriers, enabling factors, and system gaps relevant to AMR containment.

### Quality assessment

Several measures were implemented to minimize bias and enhance reliability. These included the use of predefined eligibility criteria, a comprehensive and systematic search strategy, and independent data extraction by two reviewers. Discrepancies were resolved through discussion and consensus.

Methodological quality was appraised using the Mixed Methods Appraisal Tool (MMAT), version 2018 [15]. The MMAT was selected because the review included quantitative, qualitative, mixed-methods, and implementation-focused studies. Two reviewers independently assessed methodological quality using the relevant MMAT criteria for each study design. Discrepancies were resolved through discussion and consensus. Quality assessment was used to inform interpretation of findings and identify methodological limitations within the evidence base but was not used as a basis for study exclusion, consistent with recommended scoping review methodology.

### Data synthesis

Data were synthesized using descriptive and thematic approaches [12,13]. Findings were mapped across key system domains, including governance, laboratory and surveillance capacity, workforce and infrastructure, antimicrobial stewardship, and biosafety and biosecurity practices.

A conceptual framework integrating health systems strengthening and One Health perspectives was applied to organize findings across human, animal, and environmental sectors. The synthesis focused on identifying implementation gaps, enabling factors, system bottlenecks, and evidence gaps relevant to AMR containment in Kenya.

## Results

### Study selection

The literature search identified 2,140 records, including 1,667 from bibliographic databases and 473 from registers and grey literature sources. After removal of 82 duplicate records, 2,058 records remained. Of these, 1,581 records were excluded prior to screening (985 through automation tools and 596 for other reasons), leaving 477 records for title and abstract screening.

Following screening, 331 records were excluded, and 146 full-text reports were sought for retrieval. Seventeen reports could not be retrieved, resulting in 129 full-text articles assessed for eligibility. Of these, 98 were excluded for the following reasons: lack of Kenyan relevance (n = 58), absence of biosafety, biosecurity, or IPC components (n = 21), lack of AMR focus (n = 13), and unrelated topics (n = 6).

A total of 31 studies met the inclusion criteria and were included in the final synthesis, comprising 23 peer-reviewed articles and 8 grey literature sources. The study selection process is presented in Fig 1 (PRISMA-ScR flow diagram).

### Characteristics of included studies

Of the 31 included studies, 23 were peer-reviewed journal articles and 8 were non-journal sources, including conference abstracts, technical reports, and academic theses. Most studies were conducted in Kenya, with additional evidence drawn from sub-Saharan Africa and global or multi-country analyses.

The studies covered national, sub-national, and facility levels and spanned human, animal, and environmental health sectors. Study designs included cross-sectional laboratory assessments, qualitative and mixed-methods evaluations, policy analyses, surveillance system evaluations, training and capacity-building studies, and digital health implementation reports. Detailed characteristics are presented in S3 Table, with thematic mapping provided in S1 Table. The included studies were published between 2022 and 2026 and represented a diverse range of methodological approaches, including quantitative, qualitative, mixed-methods, implementation, and policy-focused assessments.

### AMR system components assessed

The distribution of evidence across AMR system components was uneven (S2 Table). Human health systems were the most frequently represented domain (n = 27), followed by data integration and digital platforms (n = 16) and animal health (n = 14), while environmental health was the least represented (n = 8) (S7 Table). Governance and policy-related components were reported in a smaller subset of studies (n = 10). Laboratory-focused studies emphasized antimicrobial susceptibility testing (AST), quality assurance systems, biosafety practices, and laboratory information systems, with capacity generally stronger at national and reference laboratories than at lower-tier facilities [16–19]. In contrast, infection prevention and control (IPC), antimicrobial stewardship, workforce adequacy, waste management, and environmental surveillance were less consistently assessed across studies, indicating important evidence gaps, particularly at sub-national and facility levels.

### Multi-level AMR system readiness and functionality

Evidence on system readiness across levels (S5 Table) showed that national-level structures—including AMR policies, surveillance frameworks, and coordination mechanisms—were generally well established. However, implementation was often characterized by weak enforcement, fragmented coordination, and variability across sectors.

At the sub-national level, implementation of surveillance protocols and sentinel site networks was heterogeneous. Regions with referral laboratories demonstrated higher functionality, while rural and lower-resourced areas faced challenges related to limited laboratory capacity, weak referral systems, and inconsistent adherence to protocols.

At the facility level, higher-tier laboratories were better equipped with diagnostic capacity and biosafety infrastructure. In contrast, lower-tier facilities frequently lacked routine diagnostics, laboratory information systems, and quality assurance mechanisms, resulting in delays, incomplete reporting, and reduced data quality.

### One health integration across AMR surveillance sectors

Evidence on One Health integration (S6 Table) indicated that coordination between human and animal health sectors was the most developed, including pilot data-sharing initiatives and joint surveillance activities [20,21]. However, routine interoperability and coordinated reporting remained inconsistent.

The environmental health sector was notably under-represented (n = 8). While some studies described pilot environmental surveillance and research-based genomic analyses [22–24], routine environmental AMR monitoring was largely absent, and standardized protocols were lacking.

Digital platforms improved aggregation and visualization of human and animal health data but often excluded environmental data and faced interoperability limitations. Although governance structures supporting One Health integration were increasingly reported, implementation remained fragmented, particularly at sub-national levels.

### Methodological quality of included studies

Quality appraisal using the Mixed Methods Appraisal Tool (MMAT) indicated that most studies demonstrated moderate to high methodological rigor. Common strengths included clearly defined objectives, appropriate study designs, use of standardized assessment approaches, and transparent reporting of findings. Frequently identified limitations included reliance on cross-sectional designs, purposive or convenience sampling strategies, and limited longitudinal follow-up. While these limitations affected generalizability in some studies, they did not substantially compromise the overall utility of the evidence base for assessing AMR containment systems. Detailed appraisal results are presented in S9 Table.

## Discussion

This scoping review synthesized evidence from 31 studies examining biosafety, biosecurity, and AMR containment systems in Kenya, with most evidence drawn from human health systems (n = 27), followed by animal health (n = 14) and environmental health (n = 8). The findings suggest that Kenya has established several important strengths for AMR containment, including national governance frameworks, surveillance coordination mechanisms, laboratory networks, quality assurance systems, and emerging digital surveillance platforms. However, the effectiveness of these systems remains constrained by persistent implementation gaps at sub-national and facility levels. These patterns reflect broader challenges observed in decentralized health systems in LMICs [9,25,26]. By integrating evidence across governance, laboratory systems, surveillance platforms, workforce capacity, financing, and One Health coordination, this review provides a system-level perspective on how AMR containment functions in practice. Findings are interpreted through the complementary lenses of Health Systems Strengthening and the One Health framework, recognizing that AMR containment depends on interactions among governance, laboratory systems, workforce capacity, financing, surveillance, and multisectoral coordination across human, animal, and environmental health sectors.

### Laboratory and digital surveillance systems as areas of relative strength

Progress in Kenya’s AMR response is most evident in laboratory strengthening and digital surveillance. Evidence from multiple studies indicates improved adoption of AST, expansion of quality assurance systems, and gradual movement toward accreditation, particularly in national and referral laboratories [25,27,28]. Similar concentration of diagnostic capacity at higher-tier facilities has been observed across sub-Saharan Africa, reflecting structural inequities in laboratory system development [9,29].

Kenya’s investment in digital AMR surveillance platforms—including centralized repositories and One Health–aligned dashboards—positions it comparatively well within the region [7,30]. These platforms enhance data aggregation, reporting timeliness, and alignment with WHO GLASS requirements. However, evidence suggests that digitalisation alone is insufficient to address underlying system constraints. Persistent challenges in data completeness, standardization, and uptake across counties indicate that technological advances must be accompanied by investments in workforce capacity, infrastructure, and governance [27,31].

### Devolution as a structural driver of system heterogeneity

A key insight from this review is the central role of Kenya’s devolved governance structure in shaping AMR system performance. While national frameworks provide strategic direction, implementation is largely determined at county level, resulting in substantial variability in system readiness [26]. Counties with referral laboratories or established sentinel sites demonstrate stronger surveillance capacity, whereas rural and resource-constrained settings face persistent diagnostic and reporting limitations [8,25,27].

This pattern is consistent with evidence from other decentralized systems, where sub-national disparities in resources, technical capacity, and governance influence health system performance [9]. These findings underscore that AMR containment is not solely a technical challenge but also a governance and systems issue, requiring alignment between national strategies and sub-national implementation capacity.

### Incomplete operationalisation of One Health, particularly for environmental surveillance

Although One Health approaches are increasingly embedded in policy and pilot initiatives, operational integration remains partial. Coordination between human and animal health sectors has improved, supported by joint surveillance activities and emerging genomic collaborations [7,30]. Global and regional evidence similarly indicates that explicit One Health coordination can enhance surveillance coherence and system performance [32,33].

However, environmental health remains a critical gap, with relatively few studies addressing this domain (n = 8). The limited incorporation of environmental surveillance reflects broader global challenges in defining institutional ownership, standardizing methodologies, and securing sustainable financing for environmental AMR monitoring [10,34]. The absence of routine environmental data constrains understanding of transmission pathways and limits the comprehensiveness of surveillance systems. Strengthening this dimension will be essential for achieving fully integrated One Health surveillance. Sectoral integration patterns and gaps are detailed in S6 Table, while the frequency of domain coverage across studies is quantified in S7 Table.

### Workforce capacity: A key enabler with limited sustainability

Workforce development has played an important role in advancing AMR system functionality. Training initiatives have strengthened laboratory practice, surveillance competencies, and emerging genomic capabilities [35–37]. Similar gains have been reported in other African contexts [38,39].

However, these gains remain uneven and insufficiently institutionalized. Persistent shortages of skilled personnel, particularly in microbiology and bioinformatics, and inequitable distribution across counties limit system performance [9,32]. In Kenya’s devolved context, reliance on externally funded, project-based training further constrains sustainability. Embedding workforce development within national and county human resource systems will be critical for long-term capacity [40]. Workforce-related barriers and enabling strategies are summarised in S8 Table.

### Financing and sustainability as binding constraints

Financial sustainability remains a major barrier to effective AMR containment. Evidence from several studies indicates continued dependence on external funding for laboratory systems, surveillance platforms, and capacity-building initiatives [9,31]. This dependency raises concerns about the long-term viability of AMR systems, particularly at sub-national levels. Costing analyses identify laboratories as major recurrent cost drivers, including reagents, maintenance, and staffing [31,41].

In Kenya, limited integration of AMR activities into routine county budgets exacerbates these challenges [26]. These findings align with broader literature highlighting financing as a critical determinant of health system resilience in LMICs. Financing barriers and enabling approaches are summarized in S8 Table, reinforcing the need for sustainable domestic financing models aligned with county-level implementation realities.

### Bridging the “last-mile” gap: From surveillance to action

A recurring finding across multiple studies is the persistent gap between data generation and actionable use. Despite improvements in surveillance capacity and increasing availability of AMR data, translation into antimicrobial stewardship, IPC, and targeted public health interventions remains limited, particularly at sub-national and facility levels [10,28]. This challenge is not unique to Kenya; a facility-based study from Indonesia similarly demonstrated substantial discordance between commonly prescribed empirical antibiotics and local antimicrobial susceptibility patterns, underscoring the need to translate surveillance findings into locally adapted empirical treatment guidelines and stewardship decisions [42].

This “last-mile” gap reflects weak feedback mechanisms, limited analytical capacity outside national institutions, and unclear accountability structures for data use [7,9]. As a result, surveillance systems often function as data repositories rather than decision-support tools, constraining their public health impact.

These implementation bottlenecks, together with identified enabling strategies, are synthesized in S8 Table. Strengthening routine feedback loops, embedding basic analytical capacity within county health teams, and linking surveillance outputs to antimicrobial stewardship programs, locally adapted empirical treatment guidelines, and IPC performance indicators may help bridge this gap and enhance the utility of AMR surveillance systems.

### Strengths, limitations, and evidence gaps

This review provides a comprehensive, multi-level synthesis of biosafety, biosecurity, and AMR containment systems in Kenya, integrating evidence across governance, laboratory capacity, surveillance systems, workforce, financing, and One Health dimensions. The inclusion of both peer-reviewed and grey literature allowed for a broader representation of policy, implementation, and operational perspectives that are often underreported in conventional academic sources. This broad evidence base enabled assessment of AMR containment systems across multiple levels of the health system and across human, animal, and environmental sectors, providing a comprehensive understanding of system performance, implementation barriers, and opportunities for strengthening AMR containment within a One Health framework.

However, several limitations should be considered. The evidence base was dominated by cross-sectional and descriptive studies, limiting the ability to assess temporal trends, causality, and sustainability of AMR interventions [25,26]. Many studies relied on purposive or convenience sampling and were conducted in higher-level or urban facilities, which may introduce selection bias and limit generalizability to lower-tier and rural settings [8,9]. In addition, reliance on published and grey literature may introduce publication bias, as well-documented or well-performing systems are more likely to be reported [10].

Heterogeneity in study designs, assessment frameworks, and reporting standards also limited direct comparability across studies and precluded quantitative synthesis. Although methodological quality was generally moderate to high based on MMAT appraisal (S9 Table), variability in methodological rigor and reporting completeness should be considered when interpreting findings [15].

Important evidence gaps remain. In particular, environmental AMR surveillance, waste management systems, and operational antimicrobial stewardship at facility level were underrepresented in the literature [10,34]. There is also limited evidence on long-term system performance, financing sustainability, and the effectiveness of interventions at sub-national levels [9,31]. These gaps, together with identified implementation barriers and enabling factors summarized in S8 Table, highlight priority areas for future research and system strengthening.

### Implications for policy and practice

These findings indicate that Kenya’s AMR containment system is strongest at the policy and platform level but remains uneven at the point of service delivery. Progress will depend on stronger alignment between national strategies and county implementation, expanded diagnostic and quality assurance capacity beyond reference laboratories, and routine operationalization of One Health surveillance, particularly for environmental health. These insights are also relevant to other LMICs with decentralized health systems.

## Conclusion

This scoping review shows that Kenya has established a strong foundation for AMR containment through national governance frameworks, laboratory networks, and digital surveillance platforms. However, uneven county- and facility-level implementation, workforce limitations, and incomplete One Health operationalization—especially in environmental surveillance—continue to constrain system effectiveness. These findings are relevant to other LMICs with decentralized health systems seeking to operationalize integrated AMR containment strategies.

## Recommendations

Kenya should strengthen alignment between national AMR strategies and county-level planning, financing, and accountability; expand tier-appropriate laboratory capacity, specimen referral, and quality assurance systems; institutionalize workforce development and retention; operationalize One Health through routine environmental surveillance and interoperable cross-sector data systems; and strengthen feedback and analytical capacity so surveillance data inform antimicrobial stewardship and IPC. Future research should prioritize longitudinal, implementation-focused studies and standardized assessment frameworks, with greater emphasis on environmental surveillance, waste management, and sub-national system performance.

## Supporting information

S1 Checklist PRISMA-ScR checklist. Completed Preferred Reporting Items for Systematic Reviews and Meta-Analyses Extension for Scoping Reviews (PRISMA-ScR) checklist for this review. From: Tricco AC, Lillie E, Zarin W, O'Brien KK, Colquhoun H, Levac D, et al. PRISMA Extension for Scoping Reviews (PRISMA-ScR): Checklist and Explanation. Ann Intern Med. 2018;169:467–73. https://doi.org/10.7326/M18-0850. Licensed under CC BY 4.0.
(DOCX)

S1 Table Mapping of included studies by thematic focus and sector, showing how individual studies contribute to multiple AMR domains and One Health components. (DOCX)

S2 Table AMR system components assessed, summarizing evidence, key gaps, and implications across governance, surveillance, laboratory capacity, workforce, data systems, One Health integration, financing, and use of data for action. (DOCX)

S3 Table Characteristics of included studies, detailing study design, geographic scope, sectoral focus, and analytical approach to contextualize the evidence base. (DOCX)

S4 Table Assessment approaches used across studies, outlining the frameworks, tools, and methodologies used to evaluate governance, surveillance, laboratory capacity, workforce readiness, digital systems, and One Health integration. (DOCX)

S5 Table Multi-level AMR system performance, synthesizing strengths and bottlenecks at national, sub-national, facility, and cross-sectoral levels. (DOCX)

S6 Table One Health integration across sectors, focusing on operational interfaces between human, animal, and environmental health systems, including data integration and genomic surveillance. (DOCX)

S7 Table Frequency of AMR domain coverage across studies, providing quantitative counts of sectoral and thematic representation to highlight evidence concentration and gaps. (DOCX)

S8 Table Implementation barriers and enabling factors, synthesizing implementation barriers and enabling factors across governance, financing, infrastructure, workforce, data systems, One Health coordination, and use of surveillance data. (DOCX)

S9 Table Methodological quality appraisal of included studies using the Mixed Methods Appraisal Tool (MMAT), supporting interpretation of the strength and limitations of the evidence base. (DOCX)

S1 Dataset Literature search, screening, and extraction database (n = 2,058 records), containing bibliographic information, screening decisions, eligibility assessments, inclusion and exclusion outcomes, extracted study characteristics, and supporting metadata used during study selection and evidence synthesis. This dataset provides the underlying review database supporting the transparency, reproducibility, and verification of the review findings.
(XLSX)
