# Supplementary material for: Biosafety, biosecurity and antimicrobial resistance containment in Kenya: A One Health scoping review of national and subnational systems
Source: PLOS Glob Public Health. 2026 Aug 26;6(8):e0006477. doi: 10.1371/journal.pgph.0006477 (PMC13515016; doi:10.1371/journal.pgph.0006477)
Supplement: Supplementary file 3 [file pgph.0006477.s003.docx]

**Response to Reviewers**

**Manuscript Title:** *Biosafety, biosecurity and antimicrobial resistance containment in Kenya: A One Health scoping review of national and subnational systems*

**Manuscript ID:** PGPH-D-26-01526R1

Dear Editor,

We sincerely thank you and the reviewers for the careful evaluation of our manuscript and for the constructive feedback. We are grateful that both reviewers consider the manuscript technically sound and suitable for publication following minor revisions. We have carefully addressed all comments raised and revised the manuscript accordingly. A tracked-changes version has been provided to facilitate review.

Our point-by-point responses are presented below.

**Reviewer #1**

**Comment**

This manuscript has been revised and the limitations of this implementation and also this review have been addressed.

**Response**

We sincerely thank the reviewer for the positive assessment of our revised manuscript and for acknowledging that the previously raised concerns have been satisfactorily addressed.

**Reviewer #2**

**Comment 1**

Please correct the arithmetic errors in the PRISMA-ScR flow diagram...

**Response**

Thank you for identifying these inconsistencies.

We carefully re-examined the original screening database and confirmed that the underlying screening dataset was correct. The inconsistencies arose from transcription errors in the manuscript and Figure 1 (PRISMA-ScR flow diagram), which have now been corrected to ensure complete internal consistency.

The corrected values are:

- Records identified = **2,140**
- Records after duplicate removal = **2,058**
- Records removed before screening = **1,581** (985 removed through automation tools and 596 removed for other reasons)
- Records screened = **477**

These corrections are now consistent throughout the manuscript, Figure 1, and the underlying screening dataset.

**Comment 2**

Consider citing <https://pubmed.ncbi.nlm.nih.gov/41743866/> ...

**Response**

Thank you for this helpful suggestion.

After reviewing the recommended publication, we agreed that it strengthens the discussion by providing an example of the disconnect between antimicrobial susceptibility data and empirical prescribing in a comparable resource-limited setting.

Accordingly, we have incorporated the citation within the subsection **"Bridging the 'last-mile' gap: from surveillance to action"** to support our discussion on translating surveillance outputs into antimicrobial stewardship and locally adapted empirical treatment decisions.

The following reference has been added to the manuscript:

Tarigan MB, Saragih RM, Tarigan KA, Ginting F. *Antimicrobial resistance and empirical antibiotic use in diabetic foot infections: A retrospective study from Indonesia.* Narra J. 2025;5(3):e2895. doi:10.52225/narra.v5i3.2895.

As requested by the journal, we note that the reference list has been updated to include this additional citation, and subsequent references have been renumbered accordingly.

**Comment 3**

Overall, the article has strengthened considerably...

**Response**

We sincerely thank the reviewer for the encouraging assessment of our revised manuscript and for recommending its acceptance following these minor revisions. We greatly appreciate the constructive comments provided throughout the review process, which have substantially improved the quality and clarity of the manuscript.

Thank you again to the Academic Editor and both reviewers for your valuable time and thoughtful comments. We hope that the revisions satisfactorily address all remaining concerns and that the manuscript is now suitable for publication in *PLOS Global Public Health*.

Sincerely,

**Lazarus O. Odeny**
*On behalf of all co-authors*
