# Supplementary material for: Biosafety, biosecurity and antimicrobial resistance containment in Kenya: A One Health scoping review of national and subnational systems
Source: PLOS Glob Public Health. 2026 Aug 26;6(8):e0006477. doi: 10.1371/journal.pgph.0006477 (PMC13515016; doi:10.1371/journal.pgph.0006477)
Supplement: Supplementary file 4 [file pgph.0006477.s004.docx]

Table 3. S3. Characteristics of Included Studies

| **No.** | **Author (Year)** | **Country / Region** | **Study Design** | **Sample / Setting** |
| --- | --- | --- | --- | --- |
| S3-1 | [20] | Cameroon, Ethiopia, Kenya | Pilot laboratory evaluation | 11 microbiology laboratories assessed using SLIPTA and AMR Scorecard |
| S3-2 | [23] | Sub-Saharan Africa (Malawi focus) | Narrative review (One Health framework) | Human, animal, and environmental AMR and schistosomiasis research |
| S3-3 | [25] | East African Community | Qualitative policy and systems analysis | National AMR Action Plans, surveillance programs, expert inputs from 7 EAC countries |
| S3-4 | [33] | Germany | Qualitative interview study | 25 stakeholders from veterinary, research, and livestock sectors |
| S3-5 | [26] | Kenya | Training intervention (pre–post evaluation) | 13 public health scientists; national AMR surveillance repository isolates |
| S3-6 | [21] | Global | Expert workshop and consensus report | International experts in genomics, AMR, and One Health |
| S3-7 | [29] | Zambia | Cross-sectional laboratory capacity assessment | 7 faith-based hospital laboratories |
| S3-8 | [11] | Global (WHO regions) | Mixed-methods policy analysis | 60 National Action Plans, GLASS database, expert interviews |
| S3-9 | [28] | Kenya | Training intervention (pre–post study) | 13 laboratory staff from 5 county hospitals |
| S3-10 | [17] | Kenya (national and county levels) | Policy implementation review (M&E framework) | Review of NAP-AMR activities (2017–2022) across 47 counties |
| S3-11 | [31] | Kenya | Curriculum development and implementation study | >500 in-service healthcare workers trained across 19 counties |
| S3-12 | [34] | East African Community | Mixed-methods landscape assessment | National Public Health Laboratories and public genomic databases |
| S3-13 | [35] | Kenya | Cross-sectional laboratory study | 62 surface swabs from a tertiary hospital |
| S3-14 | [36] | USA & Central America | Environmental surveillance dissertation (mixed methods) | Coastal waters, sewage-impacted sites, human nasal swabs |
| S3-15 | [37] | Zambia | Cross-sectional laboratory capacity assessment | 8 purposively selected hospital laboratories |
| S3-16 | [38] | Kenya and Nepal | Diagnostic network optimization (DNO) intervention | Human and animal health laboratories and sample referral systems |
| S3-17 | [32] | Global (FAO member countries) | Tool development and systems assessment framework | AMR surveillance systems and laboratories in food and agriculture sectors |
| S3-18 | [8] | Global | Systematic review | 40 publications from 27 countries and 10 multicountry surveillance systems |
| S3-19 | [9] | Sub-Saharan Africa (14 countries) | Retrospective network analysis with facility survey | 504 bacteriology laboratories within national tiered networks |
| S3-20 | [39] | Hospital setting (country not specified) | Genomic case investigation | Placental swab culture contaminant analyzed by hybrid WGS |
| S3-21 | [40] | Central Africa (ECCAS region) | Narrative review | Microbiology laboratories involved in disease and AMR surveillance |
| S3-22 | [18] | Sub-Saharan Africa; Kenya focus | PhD thesis (cross-sectional & mixed-methods) | Clinical specimens from 4 countries; 219 health facilities in Kenya |
| S3-23 | [16] | Kenya | Cross-sectional multi-facility assessment | 219 public and private health facilities |
| S3-24 | [30] | Zambia | Cross-sectional survey | 77 layer poultry farmers |
| S3-25 | [41] | Zambia (Lusaka & Copperbelt) | Cross-sectional laboratory study | 365 pooled cloacal swabs from 77 layer poultry farms |
| S3-26 | [27] | Zambia | Mixed-methods educational intervention | 12 laboratory scientists from 5 hospitals |
| S3-27 | [24] | Global | Narrative review (One Health genomics) | Human, animal, environmental, and wildlife AMR studies |
| S3-28 | [19] | Kenya | Sentinel site laboratory quality assessment | 5 national AMR sentinel laboratories |
| S3-29 | [7] | Kenya | Digital health systems implementation study | National AMR data repository integrating human and animal health sectors |
| S3-30 | [10] | WHO African Region (11 countries) | Regional implementation review (webinar series) | National AMR focal points and laboratory networks |
| S3-31 | [22] | Nepal | Economic evaluation (bottom-up micro-costing) | 5 multisectoral surveillance sites (human, animal, food sectors) |

Abbreviations: AMR, antimicrobial resistance; AST, antimicrobial susceptibility testing; GLASS, Global Antimicrobial Resistance and Use Surveillance System; SLIPTA, Stepwise Laboratory Improvement Process Towards Accreditation; WGS, whole-genome sequencing.
