# Supplementary material for: Biosafety, biosecurity and antimicrobial resistance containment in Kenya: A One Health scoping review of national and subnational systems
Source: PLOS Glob Public Health. 2026 Aug 26;6(8):e0006477. doi: 10.1371/journal.pgph.0006477 (PMC13515016; doi:10.1371/journal.pgph.0006477)
Supplement: Supplementary file 5 [file pgph.0006477.s005.docx]

Table 4. S4. Frameworks and Tools Used to Assess AMR Surveillance Systems: Evidence and Implications

| **System Component / Focus** | **Frameworks and Tools Used** | **Key Findings / Insights** | **Implications for AMR Surveillance** |
| --- | --- | --- | --- |
| **Governance & Policy** | National AMR Action Plans (NAP-AMR); FAO-ATLASS Progressive Improvement Pathway (PIP); WHO AMR self-assessment; Quadripartite One Health Integrated Surveillance Guidance | AMR policies widely adopted, but implementation uneven; multisectoral coordination remains limited; governance gaps persist in many African settings | Weak implementation undermines coherence, multisectoral action, and sustainability of national AMR systems |
| **Laboratory Infrastructure & Diagnostics** | FAO-ATLASS laboratory module; laboratory readiness surveys; facility scoring tools; ISO 15189 accreditation assessments; OHAMRS dashboards; culture and AST methods (e.g., disk diffusion) | Reference laboratories generally better equipped; many lower-tier facilities lack culture/AST capacity, LIS, accreditation, and reliable utilities | Limits representativeness, reliability, and completeness of surveillance data; constrains population access to AMR diagnostics |
| **Workforce & Training** | Laboratory staff training programs; competency-based assessments; educational and mentorship interventions | Training improves AMR, genomics, and surveillance competencies; persistent shortages of microbiologists and limited continuing professional development | Weakens diagnostic quality and the sustainability of AMR surveillance programs |
| **Surveillance Design & Coverage** | Systematic reviews; Tricycle protocol; FAO-ATLASS surveillance module; multi-country surveillance assessments | Surveillance often urban-biased with limited population coverage; many countries lack nationally representative systems; variability in specimen sources | Weakens trend analysis, early detection, and evidence-based public health decision-making |
| **Data Management, Digitalization & Quality Assurance** | OHAMRS DHIS2 portals; digital dashboards; WHONET; laboratory information management tools; QA assessments | Digital platforms improve data reliability and visibility; gaps remain in interoperability, QA, and data reconciliation | Reduces trust, timeliness, and usability of AMR data for policy and clinical action |
| **One Health Integration** | FAO-ATLASS One Health guidance; Tricycle protocol; integrated genomic surveillance approaches; Quadripartite guidance | Adoption of One Health approaches increasing; environmental surveillance remains limited; sectoral silos persist | Constrains understanding of AMR transmission pathways across human, animal, and environmental sectors |
| **Advanced Technologies (Genomics)** | Whole-genome sequencing (WGS); hybrid sequencing approaches; bioinformatics pipelines | Genomics enables source tracing and resistance gene detection; high costs and infrastructure gaps limit routine use in LMICs | Slows scale-up of genomic surveillance and real-time AMR detection |
| **Financing & Sustainability** | Costing and economic evaluation studies; FAO-ATLASS PIP; national budget assessments | AMR surveillance largely donor-dependent; laboratories are major cost drivers; domestic financing often inadequate | Threatens long-term sustainability and scale-up of AMR surveillance systems |
| **Use of Surveillance Data for Action** | FAO-ATLASS outputs; OHAMRS dashboards; WHO AMR reports; national feedback mechanisms | Data generation increasing, but utilization remains limited; weak feedback loops and policy translation | Limits impact of surveillance on stewardship, IPC, and outbreak response |

Frameworks and tools are synthesized across multiple included studies. Detailed study-level attribution is provided in Supplementary Tables S1 and S3.
Abbreviations: AMR, antimicrobial resistance; AST, antimicrobial susceptibility testing; DHIS2, District Health Information Software 2; FAO-ATLASS, FAO Assessment Tool for Laboratories and AMR Surveillance Systems; LIS, laboratory information system; LMICs, low- and middle-income countries; OHAMRS, One Health AMR Surveillance; QA, quality assurance; WGS, whole-genome sequencing.
