# Supplementary material for: Biosafety, biosecurity and antimicrobial resistance containment in Kenya: A One Health scoping review of national and subnational systems
Source: PLOS Glob Public Health. 2026 Aug 26;6(8):e0006477. doi: 10.1371/journal.pgph.0006477 (PMC13515016; doi:10.1371/journal.pgph.0006477)
Supplement: Supplementary file 6 [file pgph.0006477.s006.docx]

Table 5. S5. Multi-level AMR System Readiness and Functionality: Evidence, Gaps, and Implications

| **System Level** | **Key Findings (Evidence from Included Studies)** | **Observed Strengths** | **Key Gaps / Bottlenecks** | **Implications for AMR Surveillance** |
| --- | --- | --- | --- | --- |
| **National / Policy Level** | Existence of NAP-AMR, national action frameworks, and alignment with IHR/JEE processes; establishment of AMR governance platforms | Strategic policies and frameworks largely in place; donor support available; multisectoral committees established in some settings | Weak enforcement, uneven implementation across regions, limited political commitment, fragmented coordination | Reduces coherence and sustainability of national AMR systems; limits strategic use of surveillance data and coordinated One Health action |
| **Sub-national (Regional / Provincial) Level** | Implementation of surveillance protocols, sentinel site networks, and regional laboratory oversight mechanisms | Some regional laboratories functional; partial urban population coverage; initial sample referral systems established | Uneven laboratory capacity; weak specimen referral and transport; rural undercoverage; inconsistent protocol adherence | Data gaps limit representativeness and early warning for emerging AMR patterns; weakens national trend analysis |
| **Laboratory / Facility Level** | Assessment of laboratory infrastructure, diagnostic capacity, AST, biosafety, and QA systems | Reference laboratories generally well equipped; availability of basic biosafety infrastructure; use of selected digital tools (e.g., WHONET, OHAMRS) | Limited culture and AST capacity at lower-tier facilities; LIS and accreditation gaps; intermittent utilities; shortages of trained staff | Restricts access to reliable AMR testing; reduces timeliness, quality, and completeness of surveillance data; constrains outbreak response |
| **One Health / Cross-sectoral Level** | Integration of human, animal, and environmental AMR data through pilot initiatives and genomic surveillance; adoption of FAO-ATLASS and Quadripartite guidance | Increasing recognition of One Health approaches; initial intersectoral coordination; integrated genomics in selected settings | Environmental, food/feed, and aquaculture sectors underrepresented; siloed operations; inconsistent data sharing | Constrains understanding of AMR transmission pathways; limits holistic policymaking and cross-sectoral interventions |
| **Data Management & Digitalization** | Use of digital dashboards, WHONET, DHIS2-based OHAMRS portals, QA and visualization tools | Improved data visibility, tracking, and analytics; partial integration across human and animal sectors | Limited interoperability; inconsistent QA and data reconciliation; delayed reporting; incomplete facility coverage | Reduces confidence in AMR data; limits timely policy and clinical decision-making; weakens evidence-based stewardship |
| **Advanced Technologies / Genomics** | Application of WGS, hybrid sequencing, and bioinformatics pipelines for source tracking and outbreak investigation | Enhanced resistance gene detection and genomic epidemiology at reference laboratories | High costs, limited infrastructure, bioinformatics expertise gaps, and poor scalability in LMIC settings | Slows routine genomic surveillance scale-up; limits real-time detection and One Health integration |
| **Financing & Sustainability** | Costing studies, donor-supported laboratory budgets, and national financing assessments | External funding enables initial implementation; laboratories identified as priority investment areas | Inadequate domestic financing; high recurrent costs; donor dependency; limited long-term sustainability planning | Threatens continuity and expansion of surveillance; constrains quality improvement and workforce retention |
| **Use of Surveillance Data for Action** | National reporting, dashboards, FAO-ATLASS outputs, and feedback mechanisms increasingly available | Improved visualization of trends and geographic patterns; some actionable insights generated | Surveillance outputs underutilized; weak feedback loops; limited translation into policy, IPC, and stewardship actions | Reduces impact of AMR surveillance on control measures and outbreak response; delays implementation of interventions |

Evidence is synthesized across multiple included studies. Detailed study-level attribution is provided in Supplementary Tables S1 and S3.
Abbreviations: AMR, antimicrobial resistance; AST, antimicrobial susceptibility testing; DHIS2, District Health Information Software 2; FAO-ATLASS, FAO Assessment Tool for Laboratories and AMR Surveillance Systems; IHR, International Health Regulations; JEE, Joint External Evaluation; LIS, laboratory information system; LMICs, low- and middle-income countries; OHAMRS, One Health AMR Surveillance; QA, quality assurance; WGS, whole-genome sequencing.
