# Supplementary material for: Biosafety, biosecurity and antimicrobial resistance containment in Kenya: A One Health scoping review of national and subnational systems
Source: PLOS Glob Public Health. 2026 Aug 26;6(8):e0006477. doi: 10.1371/journal.pgph.0006477 (PMC13515016; doi:10.1371/journal.pgph.0006477)
Supplement: Supplementary file 7 [file pgph.0006477.s007.docx]

Table 6. S6. One Health Integration Across AMR Surveillance Sectors

| **Integration Interface** | **Evidence of Integration** | **What Is Working** | **What Is Not Working** | **Implications for One Health Surveillance** |
| --- | --- | --- | --- | --- |
| **Human ↔ Animal Health** | Pilot linkage of clinical and veterinary AMR data; joint genomic investigations | Cross-sector awareness increasing; some shared protocols and reporting platforms | Data sharing inconsistent; parallel reporting systems; limited routine coordination | Weak detection of zoonotic transmission and cross-sector resistance trends |
| **Human ↔ Environmental Health** | Environmental sampling included in select research and pilot surveillance activities | Recognition of environmental AMR pathways in policy frameworks | Environmental data rarely integrated into routine surveillance; weak institutional ownership | Major blind spot for community-level and environmental transmission pathways |
| **Animal ↔ Environmental Health** | Limited overlap through research-based environmental and livestock studies | Early conceptual alignment under One Health frameworks | No routine surveillance interface; fragmented governance | Limits understanding of AMR amplification in food and ecological systems |
| **Cross-sectoral Data Integration** | Partial integration via DHIS2, OHAMRS dashboards, and shared genomic repositories | Improved visualization of human and animal data | Environmental sector largely excluded; interoperability gaps persist | Reduces operational value of One Health data for timely decision-making |
| **Cross-sector Genomic Surveillance** | Joint WGS analyses across human and animal isolates in selected settings | Strong analytical potential for source attribution | High cost, limited scale, and lack of routine integration | Genomics remains episodic rather than system-wide within One Health |
| **Cross-sector Governance & Coordination** | One Health committees and inter-agency AMR task forces established | Policy-level commitment and formal coordination platforms | Weak enforcement; inconsistent participation; environmental sector underrepresented | Coordination gaps undermine integrated surveillance and response |

Evidence is synthesized across multiple included studies. Detailed study-level characteristics and thematic mappings are provided in Supplementary Tables S1 and S3.
Abbreviations: AMR, antimicrobial resistance; AST, antimicrobial susceptibility testing; DHIS2, District Health Information Software 2; IHR, International Health Regulations; JEE, Joint External Evaluation; LMICs, low- and middle-income countries; OHAMRS, One Health AMR Surveillance; QA, quality assurance; WGS, whole-genome sequencing.
