# Supplementary material for: Biosafety, biosecurity and antimicrobial resistance containment in Kenya: A One Health scoping review of national and subnational systems
Source: PLOS Glob Public Health. 2026 Aug 26;6(8):e0006477. doi: 10.1371/journal.pgph.0006477 (PMC13515016; doi:10.1371/journal.pgph.0006477)
Supplement: Supplementary file 8 [file pgph.0006477.s008.docx]

Table 7. S7. Sectoral Coverage of Included Studies Across One Health Domains (n = 31)

| **One Health Domain** | **Evidence of Coverage in Included Studies** | **Number of Studies (n)** |
| --- | --- | --- |
| **Human Health** | Clinical laboratories, hospital-based surveillance, sentinel sites, antimicrobial susceptibility testing, and human health data platforms | **27** |
| **Animal Health** | Poultry and livestock AMR surveillance, veterinary laboratory testing, and animal-sector integration initiatives | **14** |
| **Environmental Health** | Environmental sampling of water, wastewater, soil, and related genomic or microbiological analyses | **8** |
| **Genomics** | Whole-genome sequencing, hybrid sequencing, resistance gene detection, and source-tracking analyses | **10** |
| **Data Integration / Digital Platforms** | DHIS2-based platforms, OHAMRS dashboards, WHONET, and cross-sector AMR data integration systems | **16** |
| **Governance / Policy** | National AMR Action Plans, One Health coordination structures, policy implementation reviews, and regulatory frameworks | **10** |

Counts reflect the number of included studies contributing evidence to each One Health domain. Many studies addressed multiple domains; therefore, totals exceed *n* = 31. Study-level domain mappings are provided in Supplementary Table S1.
