# Supplementary material for: Biosafety, biosecurity and antimicrobial resistance containment in Kenya: A One Health scoping review of national and subnational systems
Source: PLOS Glob Public Health. 2026 Aug 26;6(8):e0006477. doi: 10.1371/journal.pgph.0006477 (PMC13515016; doi:10.1371/journal.pgph.0006477)
Supplement: Supplementary file 9 [file pgph.0006477.s009.docx]

Table 8. S8. Implementation Barriers and Enablers of AMR Surveillance Systems (n = 31 Studies)

| **Thematic Area** | **Key Barriers Identified** | **Key Enablers Identified** | **Implications for AMR System Strengthening** |
| --- | --- | --- | --- |
| **Governance & Political Commitment** | Weak enforcement of NAP-AMR; fragmented multisectoral coordination; limited accountability mechanisms | Existence of national AMR action plans; multisectoral AMR committees; alignment with JEE and IHR processes | Governance must shift from policy adoption to operational accountability, enforcement, and sustained oversight |
| **Financing & Sustainability** | Heavy donor dependence; insufficient domestic financing; high recurrent laboratory costs; limited long-term sustainability planning | Costing and economic studies identifying laboratory cost drivers; inclusion of AMR in national planning instruments; FAO-ATLASS PIP support | Sustainable domestic financing and budget integration are essential to reduce reliance on external funding |
| **Laboratory Infrastructure & Diagnostics** | Limited culture and AST capacity in lower-tier laboratories; equipment shortages; intermittent water and power supply; low accreditation coverage | Well-equipped reference laboratories; gradual scale-up of quality management systems; availability of core biosafety infrastructure at central facilities | Expansion of infrastructure beyond reference laboratories is critical for representative and equitable surveillance coverage |
| **Workforce & Training** | Shortages of trained microbiologists and bioinformaticians; limited continuous professional development; staff turnover | Targeted laboratory and genomics training programs; mentorship and capacity-building initiatives | Workforce development must be institutionalized rather than project-based to sustain surveillance functionality |
| **Data Management & Digital Systems** | Limited interoperability; inconsistent data reconciliation; delayed reporting; partial integration of environmental data | DHIS2-based OHAMRS dashboards; adoption of WHONET; improved national visualization and reporting platforms | Interoperable, quality-assured digital systems are central to actionable and timely AMR surveillance |
| **One Health Integration** | Sectoral silos; weak environmental representation; inconsistent veterinary testing; fragmented data-sharing mechanisms | Adoption of Quadripartite guidance; cross-sector AMR committees; Tricycle protocol pilots; integrated genomic studies | Formalized cross-sector coordination and standardized environmental surveillance are required for full One Health functionality |
| **Advanced Technologies (Genomics)** | High sequencing costs; limited infrastructure; bioinformatics expertise gaps; concentration of capacity in reference laboratories | Demonstrated utility for outbreak investigation and source-tracking; hybrid sequencing approaches; research–public health collaborations | Strategic investment in regional genomic hubs may enable scalable integration into routine surveillance |
| **Use of Surveillance Data for Action** | Underutilization of surveillance outputs; weak feedback loops to facilities; limited translation into stewardship and IPC programs | Emerging dashboards enabling geographic mapping; national AMR reports; increasing policy-level awareness | Surveillance systems must strengthen feedback mechanisms to translate data into concrete public health action |

Barriers and enablers are synthesized across multiple included studies. Detailed study-level characteristics and thematic mappings are provided in **Supplementary Tables S1 and S3**.
Abbreviations: AMR, antimicrobial resistance; AST, antimicrobial susceptibility testing; DHIS2, District Health Information Software 2; FAO-ATLASS, FAO Assessment Tool for Laboratories and AMR Surveillance Systems; IHR, International Health Regulations; IPC, JEE, Joint External Evaluation; NAP-AMR, National Action Plan on Antimicrobial Resistance; OHAMRS, One Health AMR Surveillance.
