# Supplementary material for: Biosafety, biosecurity and antimicrobial resistance containment in Kenya: A One Health scoping review of national and subnational systems
Source: PLOS Glob Public Health. 2026 Aug 26;6(8):e0006477. doi: 10.1371/journal.pgph.0006477 (PMC13515016; doi:10.1371/journal.pgph.0006477)
Supplement: Supplementary file 10 [file pgph.0006477.s010.docx]

Table 9. S9. Methodological Quality of Included Studies (MMAT-based appraisal, n = 31)

| **Study (Author, Year)** | **Study Design** | **Overall Rigor*** | **Key Methodological Strengths** | **Key Limitations** |
| --- | --- | --- | --- | --- |
| [20] | Pilot laboratory evaluation | Moderate–High | Use of standardized, ISO-aligned tools; structured scoring | Small purposive sample; short pilot duration; inter-rater reliability not reported |
| [23] | Narrative review | Moderate | Integrates One Health perspectives across sectors | No primary data; potential selection bias |
| [25] | Qualitative policy analysis | Moderate–High | Triangulation of expert input and policy documents | Small purposive sample; limited generalizability |
| [33] | Qualitative interview study | Moderate | Multiple stakeholder perspectives captured | Small sample; country-specific context |
| [26] | Training intervention (pre–post) | Moderate–High | Clear intervention protocol and outcome assessment | Small sample; no control group |
| [21] | Expert workshop / consensus | Moderate | Structured expert synthesis | Opinion-based; no primary data |
| [29] | Cross-sectional laboratory assessment | Moderate–High | Multi-site structured evaluation | Small number of facilities; self-report bias |
| [11] | Mixed-methods policy analysis | Moderate–High | Triangulation of NAPs, GLASS data, and interviews | Variable data quality; subjective document analysis |
| [28] | Training intervention (pre–post) | Moderate | Standardized training and assessment | Small sample; short follow-up; no control |
| [17] | Document review / M&E assessment | Moderate | Comprehensive national coverage | Reliance on self-reported data; descriptive design |
| [31] | Curriculum development study | Moderate | Iterative, multidisciplinary design | No quantitative outcome evaluation |
| [34] | Mixed-methods genomics assessment | High | Extensive genomic and capacity evaluation | Reliance on public datasets; descriptive focus |
| [35] | Environmental cross-sectional study | Moderate–High | Rigorous sampling and AST methods | Single facility; purposive sampling |
| [36] | Longitudinal environmental surveillance | High | Spatiotemporal sampling across sectors | Regional focus; limited WGS integration |
| [37] | Cross-sectional laboratory capacity study | Moderate–High | Standardized multi-site assessment | Small sample; potential self-report bias |
| [38] | Diagnostic network optimization intervention | High | Systematic network modeling | Model outputs dependent on input data quality |
| [32] | Tool development & validation | High | Standardized assessment framework across countries | Semi-quantitative scoring; indirect evidence |
| [8] | Systematic review | High† | Comprehensive search and synthesis | Heterogeneity; variable quality of included studies |
| [9] | Multi-country lab network survey | High | Large, multi-country scope with geospatial modeling | Self-report; cross-country heterogeneity |
| [39] | Genomic case investigation | High | Rigorous genomic workflow | Single case; descriptive only |
| [40] | Regional narrative review | Moderate | Broad regional overview | Descriptive; relies on secondary data |
| [16] | Cross-sectional facility survey | Moderate–High | Structured scoring across facilities | Convenience sampling; human health focus only |
| [18] | Mixed-methods PhD thesis | High | Primary microbiological and system-level data | Sampling limits representativeness |
| [30] | Cross-sectional analytical study | Moderate | Well-defined population; robust analysis | Small sample; limited geographic scope |
| [41] | Cross-sectional poultry laboratory study | Moderate–High | Strong laboratory QA and sampling | Cross-sectional design; no molecular resistance analysis |
| [27] | Educational intervention (pre–post) | Moderate | Complete response; structured evaluation | Very small sample; no control group |
| [24] | Narrative review / perspective | Moderate | Integrates One Health genomics concepts | Non-empirical; conceptual focus |
| [19] | Sentinel site QA assessment | Moderate–High | Standardized AST and QA procedures | Limited sites; ongoing mentorship required |
| [7] | Digital surveillance system evaluation | Moderate–High | Comprehensive OHAMRS platform assessment | Descriptive system evaluation |
| WHO (2025) | Regional synthesis (webinar series) | Moderate | Multi-country experiential synthesis | No primary data collection |
| [22] | Micro-costing economic evaluation | High | Detailed bottom-up costing | Site-specific assumptions; limited generalizability |

* Overall rigor was assessed using the Mixed Methods Appraisal Tool (MMAT, 2018) and categorized as *high*, *moderate–high*, or *moderate*.
† For reviews, methodological rigor refers to review conduct, not strength of underlying evidence.
