# Supplementary material for: Biosafety, biosecurity and antimicrobial resistance containment in Kenya: A One Health scoping review of national and subnational systems
Source: PLOS Glob Public Health. 2026 Aug 26;6(8):e0006477. doi: 10.1371/journal.pgph.0006477 (PMC13515016; doi:10.1371/journal.pgph.0006477)
Supplement: Supplementary file 13 [file pgph.0006477.s002.docx]

**POINT-BY-POINT RESPONSE TO REVIEWER AND EDITOR COMMENTS**

We sincerely thank the Editor and Reviewers for their careful evaluation of our manuscript entitled “Biosafety, Biosecurity and Antimicrobial Resistance Containment in Kenya: A One Health Scoping Review of National and Subnational Systems.” Their comments were highly valuable and have substantially improved the quality, clarity, and methodological rigor of the manuscript. All comments have been carefully considered and addressed as detailed below. Line numbers refer to the clean revised manuscript.

## Reviewer 1

| **ID** | **Comment** | **Response** | **Location of revision** |
| --- | --- | --- | --- |
| R1-0 | The title is quite interesting. | We thank the reviewer for the positive assessment of the manuscript title. No revision was required. | No manuscript revision required. |
| R1-1 | Remove background subheadings and improve flow. | The Introduction was substantially revised to improve narrative flow. Internal subheadings were removed and the section was rewritten as a continuous narrative. The research gap and study objective were consolidated into the final paragraphs of the Introduction. | Introduction, lines 49–108. |
| R1-2 | Add epidemiological context. | We expanded the opening section of the Introduction to include WHO policy context, GLASS surveillance findings, and global AMR burden estimates, including the 4.95 million AMR-associated deaths and 1.27 million attributable deaths reported for 2019. This provides a stronger justification for examining AMR containment systems. | Introduction, lines 49–58. |
| R1-3 | Research gap should be at the end of the background. | The research gap and study objective were moved to the concluding paragraphs of the Introduction to improve logical progression and alignment with standard scientific writing conventions. | Introduction, lines 96–108. |
| R1-4 | Explain implementation of biosafety, biosecurity, surveillance and stewardship in Kenya. | Additional Kenya-specific context was incorporated describing national AMR governance structures, implementation of the National Action Plan, surveillance systems, stewardship coordination, laboratory strengthening efforts, and ongoing One Health coordination mechanisms. | Introduction, lines 69–95. |
| R1-5 | Is this paper intended for a seminar or symposium? | We clarified the study design and reporting framework in the Methods section, explicitly describing the work as a scoping review conducted according to Arksey and O'Malley, Levac et al., and PRISMA-ScR guidance. | Methods – Study Design, lines 112–118; response clarification only. |
| R1-6 | PRISMA diagram should be included in Methods. | The PRISMA-ScR flow diagram is now explicitly referenced within the Study Selection subsection of the Methods. | Methods – Study Selection, lines 154–162; Results – Study Selection, lines 190–202. |
| R1-7 | Sampling Technique and Sample Size not clearly explained. | References to sampling technique and sample size were removed and the methodology was reorganized around eligibility criteria, search strategy, study selection, data extraction, quality assessment and synthesis, consistent with PRISMA-ScR guidance. | Methods – Study Selection, lines 154–162. |
| R1-8 | Study Selection requires explanation of who and how. | The Methods section was expanded to describe the screening process, including title and abstract screening, full-text review, reviewer roles, independent assessment procedures, and consensus-based resolution of disagreements. | Methods – Study Selection, lines 154–162. |
| R1-9 | Search strategy insufficiently described. | The search strategy was expanded to include databases searched, grey literature sources, search terms, Boolean operators, date restrictions, and an example search string. | Methods – Information Sources and Search Strategy, lines 139–153. |
| R1-10 | Quality assessment process unclear. | The MMAT appraisal process was described in greater detail, including reviewer procedures, relevant MMAT criteria, consensus procedures, and the role of quality appraisal in interpreting findings. | Methods – Quality Assessment, lines 169–180. |
| R1-11 | Present quality assessment results. | A narrative summary of MMAT findings was added to the Results section, and detailed quality appraisal results are presented in Supplementary Table S9. | Results – Methodological Quality of Included Studies, lines 248–258; Supporting Information, lines 504–514. |
| R1-12 | Results should synthesize multiple studies. | The Results section was revised to emphasize thematic synthesis across multiple studies rather than reporting findings from individual studies in isolation. | Results, lines 203–258; Supplementary Tables S1–S8, lines 483–505. |
| R1-13 | Characteristics of included studies should be presented. | The narrative description of included studies was expanded and linked to Supplementary Table S3, providing greater detail regarding study designs, sectors, geographic coverage, and methodological approaches. | Results – Characteristics of Included Studies, lines 203–213; Supporting Information, lines 491–493. |
| R1-14 | Discussion lacks explicit study support. | Additional citations from included studies were incorporated throughout the Discussion to strengthen the evidence supporting major conclusions and interpretations across laboratory systems, digital surveillance, devolution, One Health integration, workforce capacity, financing, and use of surveillance data. | Discussion, lines 259–367. |
| R1-15 | Framework used in Discussion is unclear. | The Discussion now explicitly states that findings are interpreted through complementary Health Systems Strengthening and One Health frameworks, providing a clearer conceptual basis for interpretation. | Discussion opening paragraph, lines 265–270. |
| R1-16 | Too many limitations. | The limitations section was balanced with a stronger discussion of study strengths, policy relevance, and implications for practice and future research. The revised section first highlights the value of the broad evidence base before discussing limitations and evidence gaps. | Discussion – Strengths, limitations, and evidence gaps, lines 342–367; Implications for policy and practice, lines 368–373. |
| R1-17 | Advantages of AMR system not highlighted. | A clearer discussion of strengths was added, highlighting Kenya’s national AMR governance structures, surveillance coordination mechanisms, laboratory networks, quality assurance systems, digital surveillance platforms, and emerging genomic surveillance capacity. | Discussion opening paragraph, lines 259–264; Laboratory and digital surveillance systems, lines 271–286. |
| R1-18 | Statistical analysis not performed appropriately and rigorously. | We clarify that this study is a scoping review and therefore did not aim to perform statistical meta-analysis. Findings were synthesized using descriptive and thematic approaches consistent with PRISMA-ScR guidance and established scoping review methodology. | Methods – Data Synthesis, lines 181–188; Results, lines 203–258. |
| R1-19 | Please present the quality assessment analysis. | A narrative interpretation of MMAT findings has been added to the Results section, while detailed assessments are presented in Supplementary Table S9. | Results – Methodological Quality of Included Studies, lines 248–258; Supporting Information, lines 506–514. |
| R1-20 | Results should draw from several studies per subsection. | All Results subsections were reviewed and revised to ensure conclusions are supported by evidence synthesized across multiple studies and supplementary tables. | Results, lines 203–258; Supporting Information, lines 483–505. |
| R1-21 | Can findings be applied to Kenya despite limitations? | The Discussion, Implications, Conclusion and Recommendations sections were strengthened to explain how findings can inform policy implementation, county-level planning, and AMR system strengthening in Kenya and similar decentralized LMIC settings. | Implications for policy and practice, lines 368–373; Conclusion and Recommendations, lines 374–388. |

## Reviewer 2

| **ID** | **Comment** | **Response** | **Location of revision** |
| --- | --- | --- | --- |
| R2-1 | Search period missing from Abstract. | The Abstract now specifies that literature searches covered the period January 2022 to February 2026. | Abstract, lines 31–33. |
| R2-2 | Sampling Technique and Sample Size inappropriate for a scoping review. | The subsection was removed and relevant information was incorporated into the Study Selection process without using quantitative sampling terminology. | Methods – Study Selection, lines 154–162. |
| R2-3 | Redundant sector frequencies. | Redundant reporting of sector frequencies was removed and retained only where necessary to support interpretation of findings. The previously separate sector-frequency subsection has been removed. | Results – AMR System Components Assessed, lines 214–225; One Health Integration, lines 239–248. |
| R2-4 | Inconsistent terminology (“reports” versus “grey literature”). | Terminology was standardized throughout the manuscript and PRISMA narrative. The term “grey literature” is now used consistently where referring to non-journal sources. | Abstract, lines 31–34; Results – Study Selection, lines 190–202; Characteristics of Included Studies, lines 203–213. |
| R2-5 | Add comparative LMIC references. | Additional comparative literature from LMIC and regional contexts was incorporated throughout the Discussion, including evidence from East Africa, sub-Saharan Africa, Nepal, Zambia, and other comparable settings. These comparisons strengthen interpretation of Kenya’s experience within broader regional and global AMR implementation challenges. | Discussion, lines 274–341; References, lines 436–459. |

## Editor

| **ID** | **Comment** | **Response** | **Location of revision** |
| --- | --- | --- | --- |
| E-1 | Financial disclosure incomplete. | The Financial Disclosure statement was revised to comply with PLOS requirements, including clarification of funding sources, author affiliation, and the role of the institution in relation to the study. | Financial Disclosure section in the submission system. |
| E-2 | Data availability statement requires supporting datasets. | The Data Availability Statement was revised and the underlying review dataset has been provided as Supporting Information. Supplementary Dataset S10 contains the literature search, screening, eligibility assessment, and data extraction database (n = 2,059 records) used in this review. Together with Supplementary Tables S1–S9, these materials provide the minimum dataset required to support reproducibility of the study selection process, evidence synthesis, and review findings. | Data Availability Statement (submission system); Supporting Information, lines 482–514. |
| E-3 | Ethics statement should appear at the beginning of Methods. | The Ethics Statement was moved to the beginning of the Methods section, immediately after Study Design. | Methods – Ethics Statement, lines 119–126. |
| E-4 & E-8 | Upload separate TIFF/EPS figures. | Figure files were prepared and uploaded separately in the format required by PLOS. | Submission files (Figure 1 TIFF/EPS file). |
| E-5 | Submit Response to Reviewers document. | A detailed point-by-point response document has been prepared and submitted. | Submission documents. |
| E-6 | Submit tracked-changes manuscript. | A tracked-changes version of the revised manuscript has been prepared and submitted. | Submission documents. |
| E-7 | Submit clean manuscript. | A clean revised version of the manuscript has been prepared and submitted. | Submission documents. |
